# Polyangiitis overlap syndrome: a rare clinical entity

**DOI:** 10.1007/s00296-023-05281-x

**Published:** 2023-01-31

**Authors:** Lorenza Bruno, Martina Mandarano, Guido Bellezza, Angelo Sidoni, Roberto Gerli, Elena Bartoloni, Carlo Perricone

**Affiliations:** 1 Rheumatology, Department of Medicine and Surgery, University of Perugia, Piazzale Giorgio Menghini, 1, 06129 Perugia, PG Italy; 2 Section of Anatomic Pathology and Histology, Department of Medicine and Surgery, University of Perugia, Perugia, Italy

**Keywords:** Vasculitis, Eosinophilic granulomatosis with polyangiitis, Granulomatosis with polyangiitis, EGPA, Eosinophilia, ANCA, Anti-PR3

## Abstract

Polyangiitis overlap syndrome is a rare clinical entity comprising patients with overlapping features of more than one vasculitis, usually eosinophilic granulomatosis with polyangiitis (EGPA) and granulomatosis with polyangiitis (GPA). Few cases of polyangiitis overlap syndrome have been described in the literature, mostly associated with c-ANCA, anti-proteinase (PR)-3 positivity, a protean clinical picture characterized by vasculitis, eosinophilia and eosinophilic infiltrates in tissues and a favorable response to steroids and immunosuppressant treatments. Herein, we present a case of a 66-year-old woman with nasal obstruction, external nose deformity, sensorineural hearing loss, peripheral blood eosinophilia, high titer anti-PR3 antibodies and lung involvement. Nasal septum biopsies showed inflammatory infiltrate with eosinophilic component; histopathology of the lung demonstrated necrotizing granulomas associated with inflammatory infiltrate composed of numerous neutrophils and some eosinophils. The patient was diagnosed with polyangiitis overlap syndrome and successfully treated with cyclophosphamide. Recognizing this entity is fundamental given the distinct clinical phenotype and outcomes to therapy in the complex scenario of ANCA-associated vasculitides.

## Introduction

Polyangiitis overlap syndrome is a rare systemic vasculitis with overlapping features of more than one systemic vasculitis [1]. This systemic vasculitis cannot fit into a single category of classical vasculitis classification. There are several cases reporting patients with overlapping features, either reporting a combination of different ANCA-associated vasculitis [2] or between a specific ANCA-associated vasculitis and a large vessels vasculitis [3]. The combination between eosinophilic granulomatosis with polyangiitis (EGPA) and granulomatosis with polyangiitis (GPA) is among the most common. Indeed, both have a systemic involvement and can affect the lungs and the upper respiratory tract sometimes representing a diagnostic challenge. The approach to patients with eosinophilia and pulmonary involvement can be puzzling, and the recognition of the underlying cause when identifiable is fundamental [4].

In this context, while EGPA is characterized by peripheral blood eosinophilia, asthma and chronic rhinosinusitis [5], GPA shows pulmonary nodules without eosinophilic infiltration and usually a more severe renal disease [6]. At the same extent, the ANCA pattern is usually different in these two clinical entities, with patients with EGPA being seronegative in up to 50% of cases and presenting with anti-MPO while patients with GPA more frequently show c-ANCA, anti-proteinase (PR)-3 antibodies [5, 6]. The ANCA profile appears to be the most relevant factor determining the clinical picture [7, 8], since there are several factors that may impede the classification of a patient as GPA or EGPA, such as the lack of histological tissue since biopsies are not obtained routinely in most cases, and classification systems that, even when adopting the novel proposed criteria, may provide discrepant classification for the same patient [9–11]. Nonetheless, the identification of patients with polyangiitis overlap syndrome of EGPA/GPA is essential because the treatment modalities and prognosis are different [12]. Herein, we present a case of polyangiitis overlap syndrome and a narrative review of the literature.

## Methods

We describe the case of a 66 year-old woman who was referred to the Section of Rheumatology. The patient signed informed consent regarding publishing her data and photographs. To identify the common features of patients with polyangiitis overlap syndrome, English language articles including #polyangiitis #Wegener #Churg-Strauss #eosinophilia #granulomatosis, #ANCA, #PR3, #MPO were identified through Embase, MEDLINE, Cochrane Central Register of Controlled Trials (CENTRAL), Web of Science, Google Scholar and the Clinical trial registries of Europe and the USA published until December 2022.

## Case presentation

**Fig. 1:**
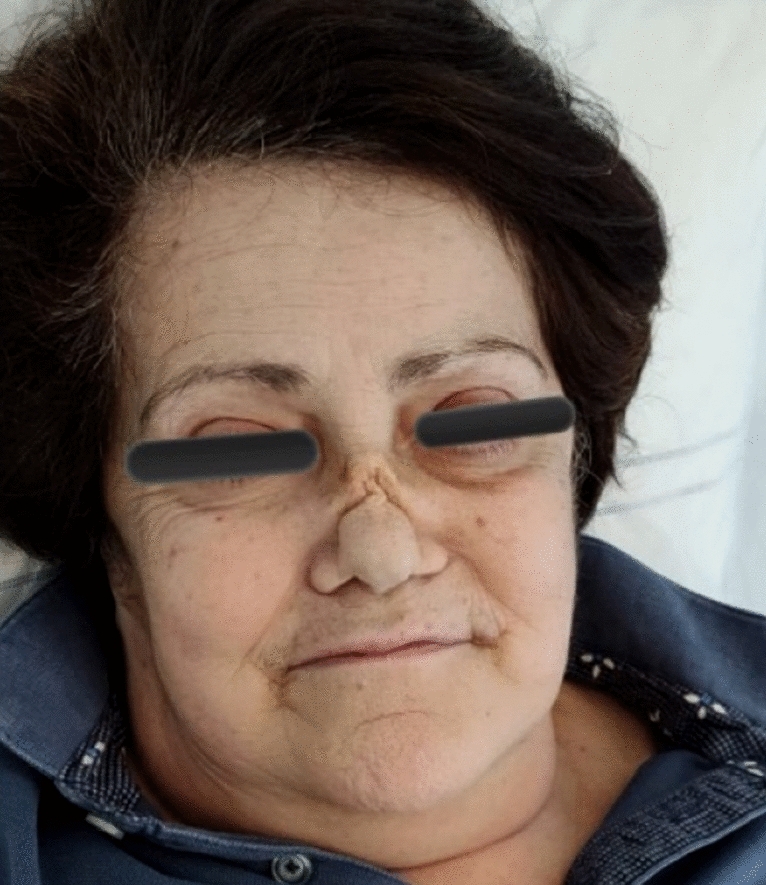
The patient presenting with saddle nose

The patient presented with progressive nasal obstruction, external nose deformity (saddle nose, Fig. 1) and sensorineural hearing loss. Laboratory findings included peripheral blood eosinophilia (1.5 × 10^9^ cells/mcl), c-ANCA titer +  +  + , high titer positivity for anti-PR3 [300 U/l, normal values (n.v.) < 10 U/l] and low titer rheumatoid factor (15.6 U/l, n.v. < 14U/l). Her past medical history was notable for allergic rhinitis and chronic rhinosinusitis, while she denied asthma and nasal polyps. Other etiologies that could account for eosinophilia, such as infections or drugs, were excluded.

**Fig. 2:**
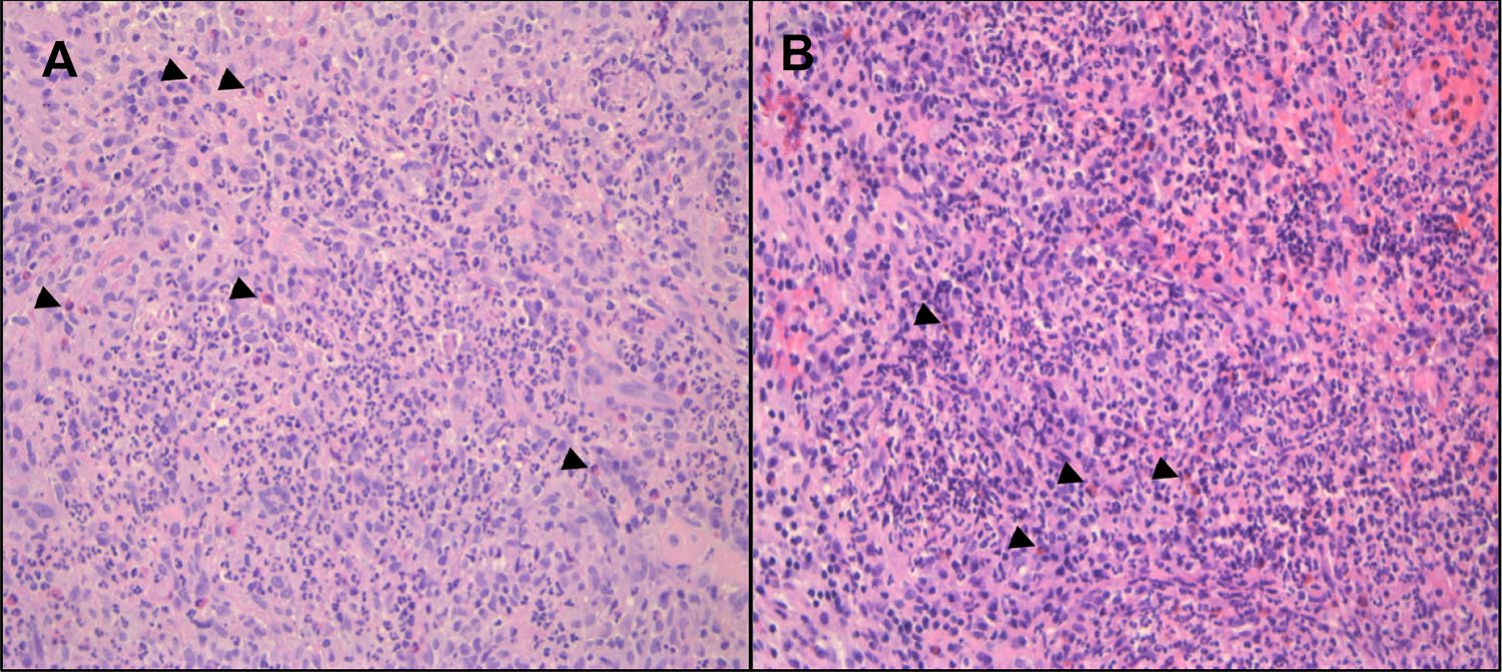
Microscopic view of nasal mucosa replaced by chronic inflammatory infiltrate with microabscesses and some eosinophils (arrow heads). **A**: February 2022, **B**: March 2022, Haematoxylin and Eosin stain, Original magnification: 200X

**Fig. 3:**
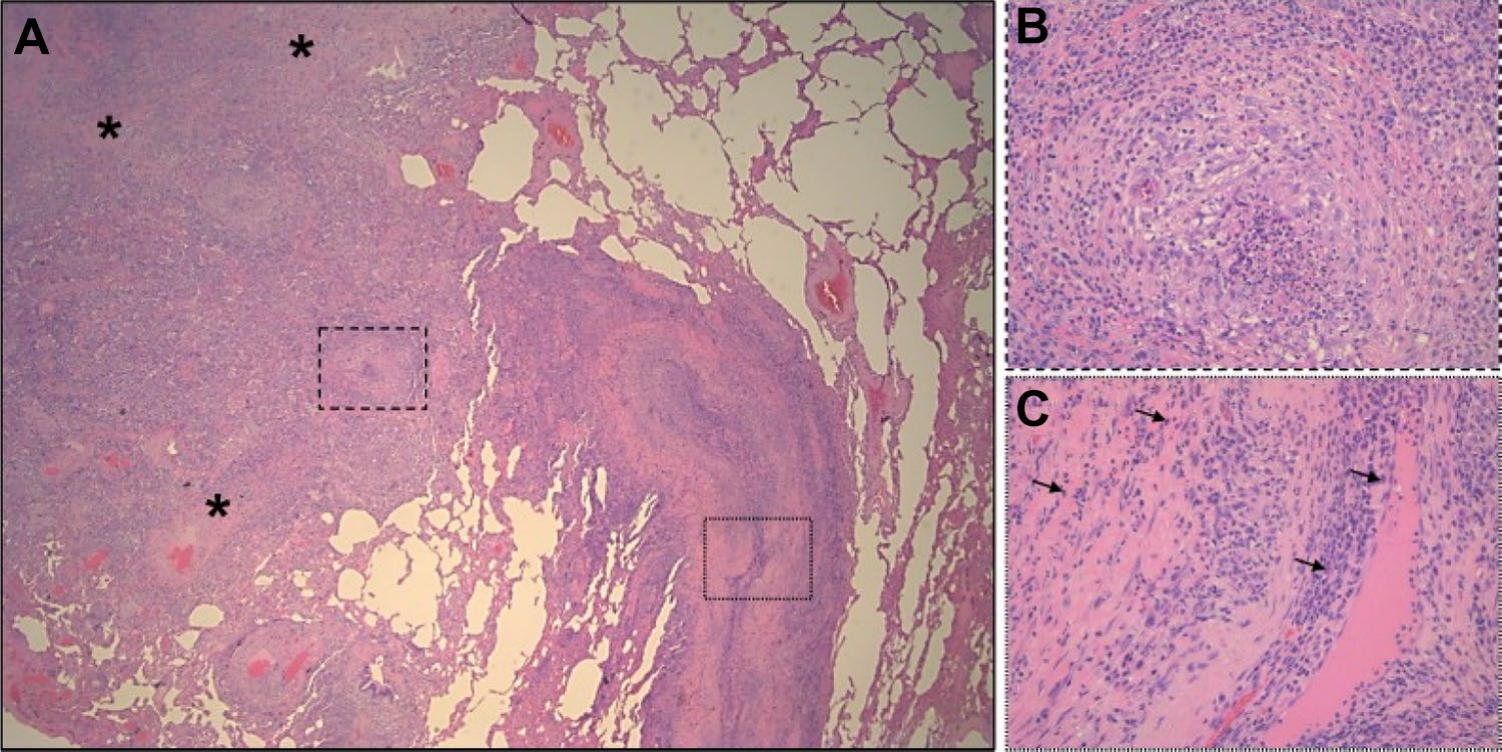
Microscopic view of lung parenchyma with altered architecture (**A**) due to the presence of necrotizing granulomatous inflammation (**B**), and areas of fibrosis (**A**, asterisks). Necrotizing vasculitis coexists (**C**), the inflammatory infiltrate which mainly consists of neutrophilic granulocytes, with eosinophils (arrows). Haematoxylin and eosin stain, original magnification: (**A**) 16X, (**B**) and (**C**) 200X

A sinus CT revealed nasal turbinate hypertrophy and the presence of a bilaterally thickened nasal mucosae with a massive swelling of the anterior portion of the nasal septum with reduced homogeneity and density due to the presence of liquefactive necrosis, determining the obstruction of the nasal vestibule. An MRI of the maxillofacial bones confirmed the CT scan findings and revealed inflammation of the mucosa covering the ethmoidal cells and left maxillary sinus, inflammation of the left sphenoid sinus and right frontal sinus. A chest radiography and CT revealed multiple bilateral nodules; the largest were localized at the right oblique fissure, adjacent to the mediastinal pleura, at the superior segment of the right lower lobe. Reticulation at lung bases without significant lymphadenopathy was also found. A (18)F-FDG PET/CT scanning showed intense increased uptake of the nasal and ethmoid sinus, at least three pulmonary hyperactive nodules in the right lung. Pulmonary tests showed an obstructive pattern. Nasal septum biopsies were performed and showed inflammatory infiltrate with predominant eosinophilic component; apparently granulomas or necrotizing angiitis were not identified (Fig. 2). To further support the diagnosis, a wedge resection of the lung was performed. Histopathology showed necrotizing granulomas associated with inflammatory infiltrate composed of numerous neutrophils and some eosinophils (Fig. 3).

The clinical, radiographic, laboratory and pathologic findings suggested EGPA/GPA overlap syndrome. Indeed, both ACR 2022 classification criteria for EGPA [5] (obstructive airway disease + 3, blood eosinophil count ≥ 1 × 10^9^/liter + 5, extravascular eosinophilic-predominant inflammation on biopsy + 2, positive test for cANCA or anti-PR3 antibodies -3, total score = 7 points) and GPA [6] (cartilagineous involvement (saddle nose deformity) + 2, sensorineural haring loss + 1, positive test for cANCA or anti-PR3 antibodies + 5, pulmonary nodules + 2, granuloma and extravascular granulomatous inflammation + 2, inflammation of nasal sinuses on imaging + 1, blood eosinophil count ≥ 1 × 10^9^/liter -4, total score = 9 points) were satisfied. The overlap is due to the combination of peripheral eosinophilia (≥ 1 × 10^9^/mcl) and necrotizing pulmonary granulomas associated with an eosinophilic infiltration (which favored EGPA), elevated serum PR3-ANCA levels and ENT involvement with nasal obstruction and saddle nose, which are much more commonly seen in patients with GPA. Sensorineural hearing loss is a clinical feature that can be observed in both conditions, although rarely in EGPA and more commonly in GPA.

**Fig. 4:**
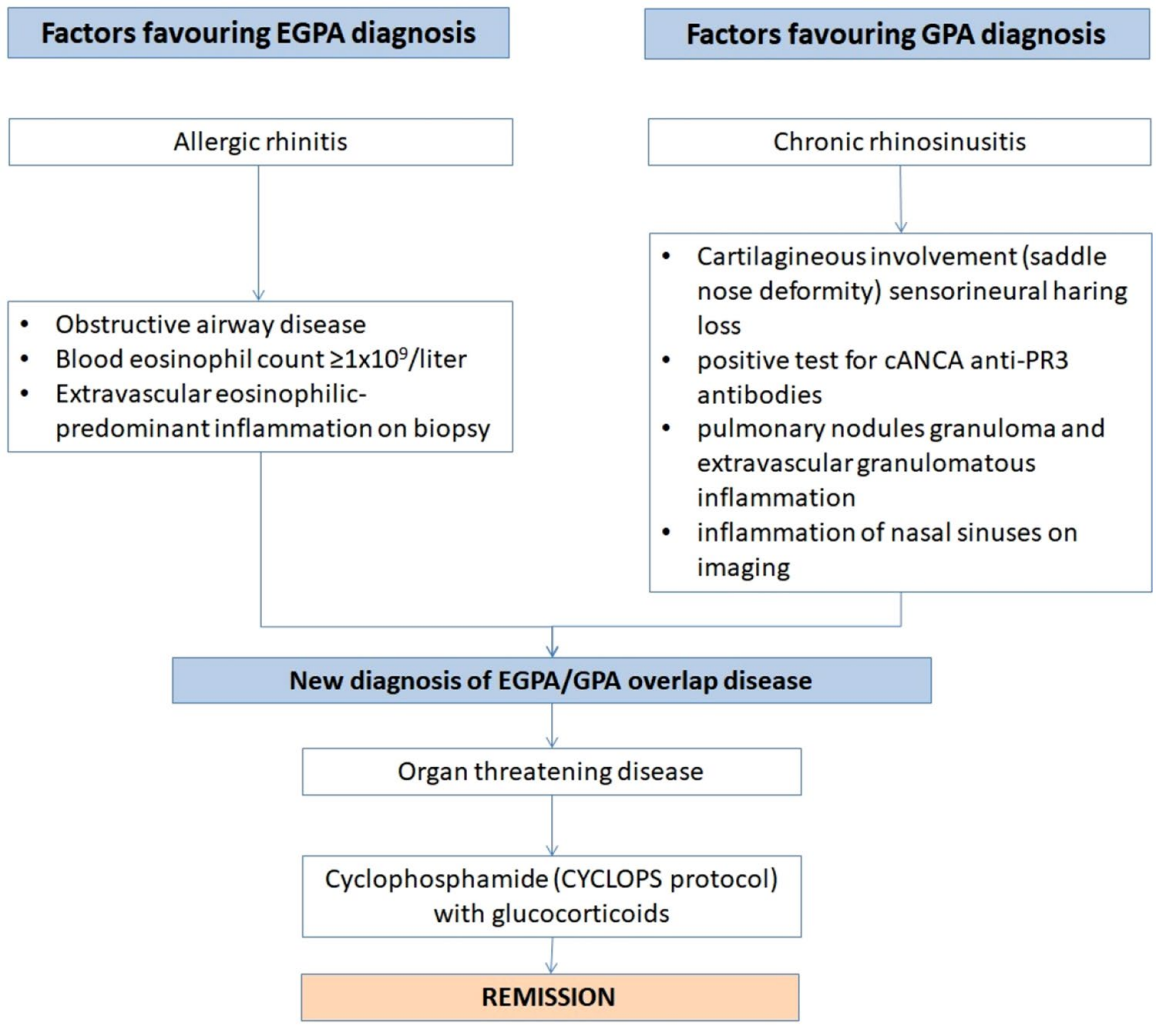
Flow-chart summarizing the main findings in this case

Given the active disease (BVAS persistent score = 1, new/worse score = 12), the patient was treated with a combination of high-dose steroids (methylprednisolone 1 g i.v. daily for three days, followed by oral prednisone, 50 mg/daily) and cyclophosphamide following CYCLOPS protocol (15 mg/kg, e.g. 1200 mg at week 0, 2, 4 and then every 3 weeks for other 4 administrations, total 8400 mg). After the second administration of i.v. steroids, hearing loss rapidly resolved and at 3 months’ follow-up the patients is in remission (BVAS persistent score = 1). A flow-chart summarizing the case is reported in Fig. 4.

## Discussion

The scenario of a patient with polyangiitis overlap syndrome is uncommon, but should be recognized since management and treatment of EGPA and GPA are different [12]. The majority of patients with EGPA may achieve remission with corticosteroids alone, while patients with GPA are usually treated with a combination of corticosteroids and immunosuppressive agents being cyclophosphamide the most commonly used. The stratification of the patients according to disease risk and severity drives the urgency of intervention. In the case of life-threatening manifestations the main difference is the largest evidence on the efficacy of cyclophosphamide and rituximab in GPA [13]. In the case of non life-treathening manifestations and the presence of generalized active disease patients will usually require therapy with cytotoxic agents including, azathioprine, methotrexate, mycophenolate mofetil or cyclophosphamide [14] with the evidence of a beneficial effect of anti-interleukin-5 monoclonal antibodies in EGPA [15].

Polyangiitis overlap syndrome can cause irreversible multiple organ failure if not treated appropriately and promptly. We identified previous cases of polyangiitis overlap syndrome reported in the literature (Table 1). Patients’ age ranged between 25 and 78 years, and most patients were women. The clinical findings were more often characterized by end organ manifestations in the lungs, kidneys, and sinuses with eosinophilia either peripheral and/or infiltrating the involved tissue. Indeed, all cases involved the lungs and over half developed alveolar hemorrhage. All patients had eosinophilia and all but one tested positive for PR3-ANCA. In all cases the patients were treated with steroids plus immunosuppressants and went into remission. Saddle nose and alveolar haemorrhage seem the most common GPA manifestations. Peripheral eosinophilia, asthma and eosinophilic infiltrate are the typical features of EGPA more frequently occurring in the overlap syndrome. When polyangiitis overlap syndrome occurs, the most severe manifestations are usually those of GPA with pulmonary involvement. It is possible that, in the spectrum of ANCA-associated vasculitis, polyangiitis overlap syndrome could be a form of PR3-positive vasculitis with associated atopy.

**Table 1:** Clinical features of cases describing EGPA and GPA overlap syndrome

Case	Sex/age	GPA	EGPA	ANCA	Treatment	Outcome
Henochowichz 1986 [20]	25 F	Arthralgia, pulmonary nodules, purpuric lesions	Rhinitis, renal biopsy-proved necrotizing glomerulonephritis, eosinophilic vasculitis, renal and nose tissue eosinophilia	N/A	CCs, CYC	Remission
Krupsky 1993 [21]	43 M	Pulmonary nodules	Marked eosinophilia in the peripheral blood, pleural effusion	c-ANCA	CCs, CYC	Remission
Potter 1999 Case 1 [22]	M 29	Nasal ulcerations, alveolar hemorrhage	Sinus congestion, peripheral eosinophilia, eosinophilic vasculitis	PR3 +	CCs, MTX, trimethoprim/sulfamethoxazole	Remission
Potter 1999 Case 2 [22]	F 30	Alveolar hemorrhage	Peripheral eosinophilia, perivascular eosinophilic inflammatory infiltrates, sinus congestion	PR3 +	CCs, MTX, trimethoprim/sulfamethoxazole	Remission
Lane 2002 Case 1 [23]	N/A	Crusting, Nasal obstruction, sinusitis, lung infiltrates	Renal biopsy proved eosinophilic infiltrate	c-ANCA	N/A	N/A
Lane 2002 Case 2 [23]	N/A	Crusting, Nasal obstruction, deafness, bloody nasal discharge	Renal biopsy proved eosinophilic infiltrate	c-ANCA, PR3 +	N/A	N/A
Lane 2002 Case 3 [23]	N/A	Lung infiltrates	Peripheral eosinophilia, renal biopsy proved eosinophilic infiltrate	c-ANCA	N/A	N/A
Lane 2002 Case 4 [23]	N/A	Pulmonary nodules	Peripheral eosinophilia, Renal biopsy proved eosinophilic infiltrate	c-ANCA, PR3 +	N/A	N/A
Lane 2002 Case 5 [23]	N/A	Lung infiltrates	Renal biopsy proved eosinophilic infiltrate	c-ANCA	N/A	N/A
Shoda 2005 [24]	F 25	Pulmonary hemorrhage, suprascleritis; granulomatous inflammation of the nasal mucosa	Paranasal sinus abnormality, eosinophilia in the blood and around the blood vessels in the biopsy samples; elevated IgE levels	PR3 +	CCs, MTX, CYC	Remission
Uematsu 2014 [25]	F 78	Nasal obstruction with epistaxis; focal panarteritis with necrotizing vasculitis and histiocytic proliferation without eosinophilic infiltration at skin biopsy, microhaematuria	Peripheral eosinophilia, asthma	PR3 +	CCs, CYC	Remission
Surendran 2017 [26]	F 45	Rapidly progressive renale failure, Proptosis of right eye, saddle nose deformity; diffuse alveolar hemorrhage	Asthma; renal biopsy proved eosinophilic infiltrate	PR3 +	CCs, CYC	Remission
Quan 2018 [27]	F 50	Cavitary pulmonary lesions	Peripheral neuropathy; long-standing asthma; chronic sinusitis; peripheral eosinophilia; elevated IgE levels, eosinophilic infiltration in the pulmonary parenchyma	**neg**	Anti-IL-5 AZA CCs	Remission

*N/A* not available, *MTX* methotrexate, *CCs* steroids, *CYC* cyclophosphamide, *AZA* azathioprine

Interestingly, only one patient was treated with anti-IL-5 agents, suggesting a predominant “vasculitic” rather than “allergic” pattern, although it should be noted that the majority of the cases were reported previous to the availability of these drugs. In this view, when considering GPA and microscopic polyangiitis (MPA), genetic associations are stronger with ANCA specificity (PR3- or MPO-ANCA) than with the clinical diagnosis [16]. Intriguingly, Lyons et al. [17] suggested that, within patients with EGPA stratified by ANCA, two genetically and clinically distinct syndromes exist. Indeed, MPO + ANCA EGPA seems to be an eosinophilic autoimmune disease more likely developing vasculitis and necrotising glomerulonephritis and an HLA-DQ association with MPO + ANCA-associated vasculitis. ANCA-negative EGPA may instead have a mucosal/barrier dysfunction origin supported by the association with barrier protein GPA33 and shared genetic architecture with inflammatory bowel diseases. In this context, a third cluster of EGPA patients may develop PR3 antibodies, while having a classic HLA class I or II-associated autoimmune disease with eosinophilic features. These patients may present specific single-nucleotide polymorphism variants such as rs141530233 and rs1042169 at the HLA-DPB1 locus which were associated with increased frequency of complementary PR3-reactive T cells [18]. This would explain the contemporary presence of anti-PR3, eosinophilia and prominent features of vasculitis. This distinction suggests important heterogeneity in the pathogenesis of the polyangiitis overlap syndrome, and highlights how this further subset of ANCA-associated vasculitis may have distinct clinical phenotype and outcomes to therapy. We shall remind that the evidence from the literature reinforces the fact that treatment strategies are tailored to the severity of the disease rather than to the ANCA phenotype [19], leading usually to a favorable outcome in patients with polyangiitis overlap syndrome.
